# Positive Youth Development in the Context of Climate Change: A Systematic Review

**DOI:** 10.3389/fpsyg.2021.786119

**Published:** 2021-11-23

**Authors:** Teresa Pereira, Teresa Freire

**Affiliations:** Department of Applied Psychology, School of Psychology, University of Minho, Braga, Portugal

**Keywords:** developmental psychology, positive youth development, climate change, adolescents and young adults, systematic review

## Abstract

Climate change has been acknowledged as one of the most significant current threats for younger generations. However, few studies have focused on climate change impacts on youth and how they can be supported. The purpose of this systematic review is to emphasize that a developmental perspective is fundamental within the interdisciplinary studies concerning climate change. Specifically, we focus our research on how the Positive Youth Development framework may inform future approaches to promote adolescents' and young adults' well-being and engagement in the context of climate change. A systematic review was conducted following Preferred Reporting Items for Systematic Reviews and Meta-Analyses guidelines. The search comprised two databases, and a total of 13 articles were finally considered eligible for review. Data were analyzed using a narrative method. The results show that the Positive Youth Development theory is not yet directly embedded in existing studies concerning adolescents and young adults in the context of climate change, but some of its principles were identified. Examples are provided of how Positive Youth Development characteristics and constructs can enhance future research, practice, and policies. We highlight this framework as an innovative and promising approach in the context of climate change.

## Introduction

### Climate Change and Youth Development

Climate change is considered one of the most critical contemporary threats (Stanley et al., 2021). Serious risks and impacts on biodiversity, ecosystems, population health and livelihoods are expected (IPCC, 2018). The most recent report from the Intergovernmental Panel on Climate Change (IPCC, 2021) has added robust evidence about the role of human influence on the current state of the climate and the human actions that can still determine future scenarios. Human beings have, thus, a triple role concerning climate change: accountable actors, victims, and agents of change. Furthermore, climate change is said to be an intergenerational issue. It compounds a challenge that opposes and transcends generations, requiring interventions and solutions currently focused on the younger population's potential (Gauvain, 2018; Sanson et al., 2018; Clemens et al., 2020).

Existing research is more focused on adults than youth (Majeed and Lee, 2017; Burke et al., 2018; Sanson et al., 2018), and most studies result from the transfer of literature on related topics (Clemens et al., 2020; Han and Ahn, 2020). Nevertheless, evidence has been collected concerning the climate change impacts on youth development. Adolescents and young adults are particularly vulnerable to climate change since several of their present and future life dimensions may be negatively affected: the surrounding socioeconomic conditions, their security, well-being, physical and mental health, personal and interpersonal development, and sense of future (Ojala, 2015; Clayton et al., 2017; Sanson et al., 2018; Han and Ahn, 2020). Overall, three types of impacts from climate change have been described: (1) direct effects of extreme events, (2) indirect effects through disruptions to social, economic, and environmental determinants of physical and mental health, and (3) indirect effects as distress and anxiety about the future due to this global environmental threat (Fritze et al., 2008; Clemens et al., 2020).

Regarding the first type of impact, the literature provides evidence for poorer mental health, changes in behavior, development, memory, executive function, decision-making, and scholastic achievement in children and youth due to exposure to extreme events (Clayton et al., 2017; Clemens et al., 2020). From a developmental point of view, this is explained by their physiological immaturity and significant dependency on parental physical, emotional, and social well-being (Sanson et al., 2019). Thus, the most disadvantaged children and adolescents may be particularly affected with long-term educational and economic consequences (Clemens et al., 2020).

Secondly, significant changes in social and environmental determinants of health and development have been noticed due to climate change (Watts et al., 2021). For instance, rising temperatures, droughts, floods, and severe storms have been associated with malnutrition, diminished quality of life, psychological distress, elevated interpersonal and intergroup conflict, inflated negative affect, or compromised sense of belonging, while also affecting outdoor recreational opportunities (Evans, 2019). This is particularly relevant for youth since nature constitutes a developmental resource, benefiting physical, cognitive, social, and emotional outcomes (Bowers et al., 2021). The opportunity of growing up in a supportive, enabling, and secure environment may be seriously compromised.

Thirdly, climate change impacts are not restricted to youth already experiencing distress from extreme events since indirect encounters with climate change may also arise from exposure to media coverage, educational resources, or interpersonal interactions (Swim et al., 2011; Ojala, 2015; Clemens et al., 2020). A recent international study on climate anxiety found that 60% of the respondents reported feeling “very” or “extremely” worried about climate change, and nearly half (45%) asserted that their feelings about climate change were negatively affecting their daily lives (Hickman et al., 2021). Not surprisingly, youth climate strikes have scaled worldwide in the last years, claiming climate action (Sanson et al., 2019; Han and Ahn, 2020). The academic community has justified and supported these initiatives, with thousands of scientists stating that countries fail to act on climate change (Warren, 2019). In addition, youth potential on helping to solve this global challenge has been increasingly acknowledged (Kleinert and Horton, 2016; Sawyer et al., 2018).

### A Developmental Perspective on Climate Change

Research shows the importance of investing in this age period from a developmental point of view. For instance, a systematic review on youth perceptions about climate change reported that levels of belief, concern, and willingness to take some actions declined from younger to older youth and then expectably raised as they become young adults (Lee et al., 2020). This has been previously named the “adolescent dip” in environmental attitudes and behaviors (Olsson and Gericke, 2016). Psychological and emotional development through adolescence points to the progressive maturation of the brain, from early adolescence to middle and late adolescence, being the final phase of the adult brain organization nearby young adulthood (Patton et al., 2016). This would mean the achievement of greater future orientation and the cumulative capacity to weight the long-term impacts of decisions. However, adolescents are developing in a rapidly changing world (Dahl et al., 2018) and the interaction of multiple factors may determine climate action. Thus, the positive development of cognitive, affective, self-regulatory capacities, and an adult identity in close interaction with an increasingly complex social set is crucial to shaping conscious consumers and active and adapted citizens (Patton et al., 2016; Dahl et al., 2018). Consequently, adolescence has been acknowledged as a sensitive period for learning and shaping behaviors, providing opportunities for pivotal influences on developmental trajectories (Dahl et al., 2018).

Thus, a developmental perspective is fundamental within the interdisciplinary effort of understanding the impact of climate change on youth and how they can be supported (Gauvain, 2018; Sanson et al., 2018; Allen, 2020). Historically, developmental psychology has been focused on promoting well-being and enhanced life chances for all (Lerner et al., 2002; Lerner et al., 2005; Lerner et al., 2012). Specifically, this scientific branch reunites the expertise for examining developmental pathways of risk, resilience, and well-being; studies the causes of human behavior and how to change it, and; offers models and interventions for developing protective skills, managing negative emotions and fostering engagement of adolescents and young adults as future and current agents of change (Petersen and Verma, 2018; Sanson et al., 2018; Han and Ahn, 2020). In addition, developmental researchers draw on the bioecological systems perspective (Bronfenbrenner and Morris, 2006) for understanding the complex interplays between a changing environment and individual development. They also privilege models that incorporate adolescents' search for autonomy, novelty, and opportunities to demonstrate courage and responsibility (Sanson et al., 2018).

### Contributions From Positive Youth Development Framework

Positive Youth Development (PYD) has risen on the intersection of developmental and bioecological models, drawing particularly on the concepts of plasticity of human development and adaptive developmental regulations (Lerner et al., 2002; Leman et al., 2017; Lerner and Chase, 2019; Shek et al., 2019). Combining these concepts suggests that there is potential for promoting positive changes throughout development and that mutually beneficial individual-context relations lead to positive individual and societal development (Lerner et al., 2005a, 2006). Thus, PYD is related to developmental experiences conducive to youth thriving and attaining adult potential and well-being (Lerner et al., 2000, 2002; Benson and Scales, 2009). Accordingly, PYD approaches are broadly designed to build skills, foster agency, build healthy relationships, strengthen the environment, and transform systems (Catalano et al., 2019). Several PYD models have been proposed in the scientific literature, such as social-emotional learning (Zins and Elias, 2007), Benson's model on external and internal developmental assets (Benson et al., 2011), and Catalano's 15 PYD constructs (Catalano et al., 2002). However, one of the most prominent and empirically supported frameworks (Arnold and Silliman, 2017) is the Five Cs Model of Positive Youth Development (Lerner et al., 2005b). According to this model, thriving reflects the manifestation of the Five Cs (competence, confidence, connection, character, and caring or compassion) over time, leading to an additional sixth C that consists of youth contribution to their positive development and healthier surrounding contexts, such as family, community, and civil society. Nevertheless, the lack of a shared set of constructs among models remains a common vulnerability identified through PYD literature (Tolan, 2014; Ciocanel et al., 2017; Leman et al., 2017; Lerner et al., 2018; Shek et al., 2019). Thus, a recent systematic review (Catalano et al., 2019) has proposed the integration of constructs from different models, organizing PYD constructs by four domains: (1) assets—exposure to education or training, interpersonal skills, recognizing emotions and self-control; (2) agency–positive identity, self-efficacy, ability to plan, perseverance, positive feelings about the future; (3) contribution–engagement in civil society and with adults, and; (4) enabling environment–bonding, prosocial opportunities, support, prosocial norms, values, and recognition, gender-responsive, physical and psychological safety.

PYD theory has been claimed to be a valuable approach for preparing adolescents and young adults for the realities of climate change (Sanson et al., 2018, 2019; Olenik, 2019). Sanson et al. (2019) found some congruence between the characteristics that will be most useful for the next generation to adapt successfully in the context of climate change and those included in models of positive development. International donors have demonstrated a high interest in PYD, prioritizing this framework to answer global issues and challenges (Olenik, 2019). Also relevant is PYD acknowledgment within other crises. After the 2008–2009 economic recession, an intervention based on PYD principles has successfully ensured youth opportunities to be heard, empowered as change agents, and engaged in meaningful decisions (Frasquilho et al., 2018). During the Covid-19 pandemics, PYD approaches have been highlighted as the pathway forward, considering that these promote safe and structured contexts, developmental relationships with caring adults, skill-building opportunities, and chances for authentic leadership (Arnold, 2020). Concerning climate change, evidence also shows that opportunities to engage in meaningful actions may benefit well-being, as this gives the sense that something is being done (Clemens et al., 2020; Nielsen et al., 2021; Sanson and Bellemo, 2021; The Lancet Child and Adolescent Health, 2021). Furthermore, environmental action and behaviors that reflect a concern for the environment have been identified as indicators of positive development (Moore and Halle, 2001; Gomez-Baya et al., 2020).

Considering the potential relevance of positive youth development approaches in the context of climate change, this paper is focused on a systematic review of existing literature related to this topic. We found that published reviews on PYD have not identified studies related to climate change (Catalano et al., 2002, 2019; Roth and Brooks-Gunn, 2003; Lapalme et al., 2014; Sancassiani et al., 2015; Ciocanel et al., 2017; Franco and Rodrigues, 2018; García-Poole et al., 2019; Waid and Uhrich, 2019). Only a program focused on promoting environmental activism (Johnson et al., 2007), but not specifically climate change, was mentioned by Curran and Wexler (2017). Thus, we intend to explore and further advance how Positive Youth Development theory is integrated within climate change literature. We aim to provide up-to-date and comprehensive contributions from the PYD framework for future research, interventions, and policy recommendations. As a result, we expect to highlight the unique perspective and potential benefits of a developmental approach to research on adolescents and young adults in the context of climate change.

## Research Aims

We intended to identify existing studies on the interface of Positive Youth Development constructs and climate change to gather, synthesize and enrich the current empirical evidence about the potential of PYD approaches in the context of climate change. Regarding the PICOS framework (Liberati et al., 2009), we searched for studies focused on youth (population), promoting constructs related to positive youth development in the context of climate change (outcomes) and with an empirical basis (study design). No specific interventions or comparisons were required. We, thus, considered the following research questions:

- How is Positive Youth Development addressed in empirical studies relating adolescents and young adults to climate change?
- Which PYD constructs are included in these empirical studies?
- How do these studies' outcomes inform future approaches to promote positive youth development in the context of climate change?

## Method

### Study Design and Search Strategy

A systematic review was conducted following the 2009 Preferred Reporting Items for Systematic Reviews and Meta-Analyses (PRISMA) guidelines (Liberati et al., 2009; Moher et al., 2009). Literature was searched using a protocol previously designed. The search was concluded on 4th August 2020, using Scopus and Web of Science databases, and the period of publication was not limited. Considering the wide scope of the positive youth development construct, this review draws in what has been the search strategy in previous systematic reviews concerning PYD (Sancassiani et al., 2015; Catalano et al., 2019), considering a wide range of search terms to best capture related studies. Combinations of different terms were tested through preliminary searches, which informed the selection of final search terms, considering target population, climate change and PYD related terms, and type of study or intervention. The search strings were combined according to the databases, considering “abstract, title and keywords” search in Scopus and “topic” search in Web of Science. As recommended by PRISMA guidelines, we illustrate this process by providing as an example the full electronic search strategy for one of the databases (see Table 1).

**Table 1:** Search strings on Web of Science.

TOPIC: (youth* OR adolescen* OR teen* OR “young people” OR “young adult*” OR “early adult*” OR “emergent adult*”) AND TOPIC: (“climat* chang*”) AND TOPIC: (“positive youth development” OR “youth development” OR “positive development” OR “optimal functioning” OR “optimal development” OR “optimal experience” OR thriv* OR flourish* OR “positive behavior*” OR “prosocial behavior” OR “positive identit*” OR bonding OR “positive relation*” OR “positive environment” OR “climat* action” OR “engagement” OR contribution OR participation OR involvement OR agency OR “self-efficacy” OR “self-determination” OR resourc* OR skill* OR competenc* OR capacit* OR asset* OR “resilien*” OR strengh* OR coping OR “subjective well-being” OR “psychological well-being” OR “satisfaction with life” OR “life satisfaction” OR “quality of life” OR “life quality”) AND TOPIC: (impact* OR project OR program OR intervention OR outcom* OR evaluation OR result* OR research OR framework OR model OR strateg*) Refined by: RESEARCH DOMAINS: (SOCIAL SCIENCES OR ARTS HUMANITIES) AND LANGUAGES: (ENGLISH) AND TYPES OF DOCUMENT: (ARTICLE)Timespan: All years. Databases: WOS, CCC, DIIDW, KJD, MEDLINE, RSCI, SCIELO

The inclusion criteria were:

(1) peer-reviewed journal articles,
(2) articles in English only,
(3) articles from social sciences, psychology and arts, and humanities,
(4) empirical studies,
(5) studies relating adolescents and young adults with climate change and including at least one construct of positive youth development as described by Catalano et al. (2019), and
(6) studies related to adolescents and young people ranging from 10 to 24 years old.

The exclusion criteria were:

(1) studies not focused on the specific issue of climate change (e.g., studies focused on the broader issue of sustainable development) or developed around a specific component related to climate change but without integration in the global issue of climate change (e.g., energy consumption, water-saving, extreme weather events, and natural disasters),
(2) studies that include adolescents and young people as part of a larger sample,
(3) studies focused on describing youth knowledge, attitudes, or perceptions or in curriculum development, and
(4) studies without detailed information on the method and results.

### Screening and Study Selection

**Figure 1:**
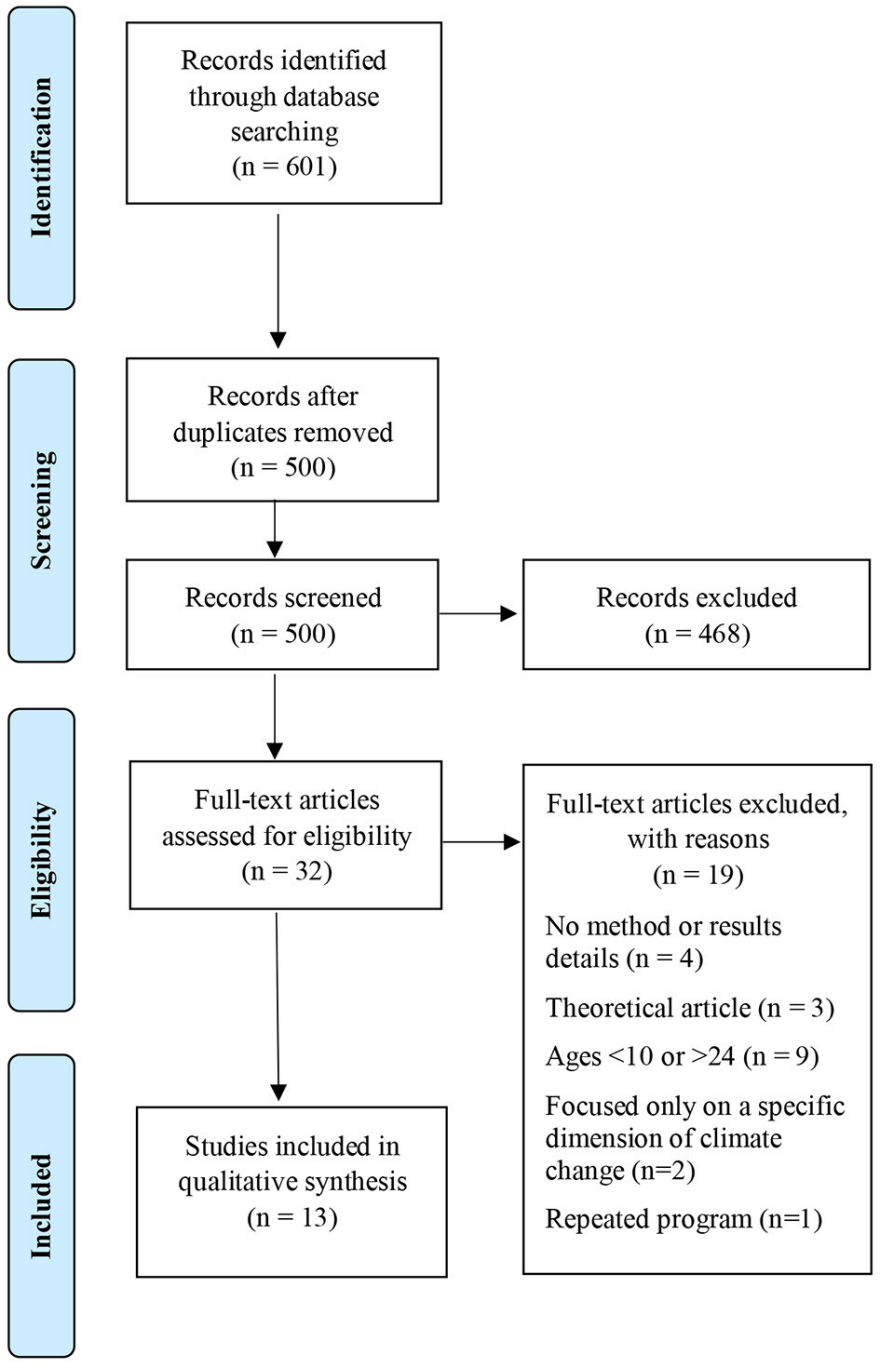
Selection of eligible articles (see submitted figure).

Considering all the search results, a total of 601 records, 453 from Web of Science, and 148 from Scopus, we independently screened the retrieved articles. After duplicates removal, the title, abstract, and keywords were scanned to determine which studies should be assessed further. A significant number of studies were not considered because climate change was not the focus as defined in the exclusion criteria. All potentially relevant articles were then analyzed as full text. Any disagreements about whether to include studies were resolved without the need of a third party. A total of 13 articles were finally considered eligible for review (see Figure 1).

### Quality Assessment

Considering the included studies' heterogeneity, we used the Quality Assessment for Diverse Studies tool (QuADS, Harrison et al., 2021), a refined version of the Quality Assessment Tool for Studies with Diverse Designs (QATSDD, Sirriyeh et al., 2012). QuADS is used to determine the methodological and reporting quality and transparency of multi- and/or mixed-methods studies when included in systematic reviews. This appraisal tool is comprised of 13 items, which are scored using a four-point scale (0–3), with a maximum possible of 39 points. These items include the following content: (1) theoretical or conceptual underpinning, (2) research aims, (3) research setting and target population, (4) appropriateness of study design to address research aims, (5) sampling appropriateness, (6) data collection tools rationale, (7) appropriateness of data collection tools to address research aims, (8) data collection procedure, (9) recruitment data, (10) analytic method, (11) appropriateness of analytic method to answer research aims, (12) involvement of stakeholders, and (13) strengths and limitations. Two reviewers independently scored the selected papers. The included studies' total rates ranged between 21 and 32 points. The lowest scores were related to items concerning sampling appropriateness, recruitment data, stakeholder involvement, critical discussion of strengths and limitations, and rationale for choosing data collection tools. This appraisal reflects some limitations in the reporting of studies rather than a judgment of the studies' quality, and therefore, none of the studies was excluded. A weighted Cohen's kappa (Cohen, 1968) of 0.86 was obtained for interrater reliability.

### Data Extraction and Synthesis

A data extraction matrix (see Table 2), developed by the research team according to the aims of this study, was used to collect the data from the included articles. The information extracted was comprised of the following aspects: (a) study characteristics, such as citation, authors affiliation, brief description, study design, participants, country, setting, and (b) specific data concerning research questions, namely PYD framework mentions, PYD constructs targeted and main results related to PYD. The retrieved data was analyzed using a narrative method. First, a preliminary synthesis of findings of included studies was developed using the data comprised in the aforementioned matrix. Secondly, the authors explored varying characteristics between studies, while also grouping findings considered conceptually similar. At last, the synthesis process was critically discussed, and the necessary adjustments were made. For the systematization of the PYD constructs, we acknowledged the categorization presented by Catalano et al. (2019), as previously mentioned.

**Table 2:** Matrix of included articles.

**Study characteristics**						**Research questions**		
**References**	**Brief description**	**Study design**	**Participants**	**Country**	**Setting**	**PYD framework mentions**	**PYD constructs included**	**Outcomes**
Bentz and O'Brien (2019)	This study explores how Art for Change project, which challenges students to adopt a sustainable behavior for 30 days and develop an art project reflecting this experience, can contribute to engage youth in individual and systems change in a changing climate	Program Exploratory study with a multi-method approach	24 students, aged 16–18	Portugal	School Home	None, but similarly the study addresses both individual and systems change	- Critical thinking - Sense of empowerment - Sustainable behaviors	Increased climate change awareness and critical thinking; New insights about own values, beliefs, emotions, and relationships to resources; Increased sense of empowerment but also some feelings of disempowerment; Influence on family and friends and continued behavioral change
Bissinger and Bogner (2018)	This study aims to put in practice an environmental literacy model promoting knowledge, environmental attitudes, and pro environmental behavior, by implementing an intervention in a botanical garden	Intervention Quantitative method and test-retest group	283 students; groups mean age 15.8 and 16.2	Germany	Botanical garden	None	- Inclusion of Nature in One's Self (as a component of attitudes) - Pro-environmental behavior intentions	Small but significant increase on the Inclusion of Nature in One's Self; Subtle but significant increase on self-reported general ecological behavior; No significant effects on test-retest group
Busch et al. (2019)	This study explores the influence of cognitive and psychosocial variables on youth's climate change-related behavior, to create an empirically supported theoretical model for youth's choice to take action to mitigate climate change	Model Quantitative study	453 middle and high school Students (ages not specified)	USA	School	None	- Efficacy -Social norms -Self-reported pro-environmental behavior specific to climate change	Social norms were the strongest direct predictor of behavior; Efficacy was a significant direct predictor of behavior; Efficacy partially mediated the effects of social norms on behavior
Deisenrieder et al. (2020)	This study analyzes, along one school year, if single components of climate change awareness differentiate between project k.i.d.Z.21-participants who have and those who have not been involved in Fridays for Future (FFF)	Program Mix-method approach	169 students; aged 11–16	Austria and Germany	School and out of school (high alpine setting) within K.i.d.Z.21 and out of school within Fridays for Future	None	- Sense of responsibility, self-efficacy, and locus of control (as part of attitudes) - Climate friendly behavior	Both groups showed a significant increase in self-efficacy and locus of control after intervention; Climate friendly behavior values raised for both groups; Higher means of some constructs were detected among FFF participants but most items of climate change awareness could be enhanced by the learning intervention
Flora et al. (2014)	This study evaluates the climate science knowledge, beliefs, attitudes, behavior and communication impact of a 1-h entertainment education high school assembly program	Program Quantitative method	1,241 students from high school (age not specified)	USA	School	None	- Positive engagement including self-efficacy and behavioral intentions - Conservation behavior	Students' positive engagement in climate change and most short-term behaviors increased significantly Effect sizes were largest for two measures of positive engagement (beliefs and self-efficacy) and remained unchanged in post-assembly measurement; The behavior most influenced by the assembly was interpersonal discussion with parents and with friends about climate change
Hu and Chen (2016)	This study explores if place-based inter-generational communication can contribute to changes in behavioral intentions, through a 30-min lecture and 30-min focus group with local seniors within a climate change educational program	Program Mixed methods approach with a control group	1,168 adolescents, aged 10–13	China	School	None	- Behavioral intention of mitigation - Perceived behavioral control - Subjective norms - Place attachment	Communication with seniors increased perceived behavioral control, subjective norm, place attachment, and mitigation intention; Changes in perceived behavioral control, subjective norms, and place attachment were strong predictors of changes in intention; Perceived behavioral control, subjective norms and place attachment were significant mediators between communication experience and mitigation intention
Ojala (2012a)	The aim of this study was to explore how Swedish 12-year-olds cope with climate change and how different coping strategies relate to environmental engagement and well-being	Exploratory study Quantitative questionnaire	293 students (mean age 12)	Sweden	School	None, but similarly the study is based on the premise that how people cope with climate change threat could be important for both engagement and psychological well-being	- Coping - Environmental efficacy and pro-environmental behavior as part of environmental engagement - Optimism concerning climate change	Problem-focused coping and meaning-focused coping were positively related to environmental efficacy, pro-environmental behavior, optimism concerning climate change, and a sense of purpose; Problem-focused coping had a positive relation to general negative affect; Meaning focused coping associated to life satisfaction and positive affect; De-emphasizing the seriousness of climate change, a kind of emotion-focused coping, was negatively associated to negative affect; Meaning focused coping, optimism and a sense of purpose seems to buffer highly problem-focused children from a high degree of negative affect
Ojala (2012b)	The purpose of this study was to explore how different age-groups of Swedish young people cope with worry and promote hope in relation to climate change	Exploratory study Qualitative and quantitative approach	90 late childhood/early adolescence (mean age 11.7); 146 senior high school (mean age 16.4); 112 young adults (mean age 22.6)	Sweden	School and university	None	- Hope - Coping	In all three age-groups, hope was primarily evoked by different meaning-focused strategies; Worry about climate change was most commonly regulated by distancing strategies (higher in children) or by problem-focused strategies
Ojala (2013)	The aim of this study was to investigate how Swedish adolescents cope with climate change and how different coping strategies are associated with environmental efficacy, pro-environmental behavior, and subjective well-being, comparing the results with a previous study with 12 years old	Exploratory study Quantitative approach	321 adolescents (mean age 17.2 years)	Sweden	School	None, but similarly the study is based on the premise that how people cope with climate change threat could be important for both engagement and psychological well-being	- Coping - Environmental efficacy and pro-environmental behavior as part of environmental engagement - Optimism concerning climate change	Problem-focused coping and meaning-focused coping had significant positive associations with environmental efficacy, and pro-environmental behavior; Only meaning-focused coping had a positive relation to optimism concerning climate change; De-emphasizing the threat, a kind of emotion-focused coping, had negative relations to environmental efficacy and pro-environmental behavior; In contrast to 12-year-olds, neither meaning-focused coping nor optimism buffered against negative affect in highly problem-focused adolescents
Ojala and Bengtsson (2019)	The aim of this study was to examine how coping with climate change among Swedish adolescents relate to pro-environmental behavior, as well as to communication patterns with parents and friends about societal and environmental issues	Model Quantitative method	705 senior high school students (mean age 18)	Sweden	School	None	- Coping - Communication - Reported pro-environmental behavior	Problem-focused coping and meaning focused coping had significant positive relations with pro-environmental behavior; Positive communication patterns with mother, father, and friends had significant positive relations, ranging from weak to medium strength, to problem- and meaning-focused coping
Sayal et al. (2016)	This study analyzes how specific components (an international exchange between a developed country and a developing country and environmental justice speakers) of an environmental program foster systems thinking and engagement in collective environmental action	Program Qualitative approach	82 participants in the program and 34 in the interviews (mean age 21 years old)	Bangladesh, Canada and India	University	None	- Systems thinking - Environmental engagement	Better understanding of environmental issues at a cognitive and emotional level; Increased capacity for systems thinking; Renewed motivation or intention to act for the environment or engaging in collective environmental action or in individual-level personal environmental action
Stevenson and Peterson (2015)	This study examined how climate change hope, despair, and concern predict pro-environmental behavior	Exploratory study Quantitative approach	205 sixth graders, 432 seventh graders, and 835 eighth graders, aged 11–15	USA	School	None	- Hope - Pro-environmental behavior	
Trott (2019)	This study aims to understand how a 15-week after-school program, “Science, Camera, Action!” facilitated participants constructive climate change engagement	Program Participatory action research method	55 students, aged 10–12	USA	Community-based youth development organization	None, but mentions the importance of looking at young people as agents of change, rather than focusing on their vulnerabilities	- Sense of agency - Constructive climate change engagement	Learning about climate change strengthened children's motivation for action, and their participation in youth-led action projects empowered their sense of agency

## Results

### Characteristics of Included Studies

All studies were published in the last decade and six within the previous 2 years. The authors' affiliation reveals that half of the studies are co-authored by researchers from both Life Sciences and Human and Social Sciences. Studies were conducted mainly in North America and Europe. A significant part of the studies took place in schools or universities. Two were conducted in more than one context (Bentz and O'Brien, 2019; Deisenrieder et al., 2020), and two included a natural setting, namely a botanical garden and a high alpine experience (Bissinger and Bogner, 2018; Deisenrieder et al., 2020). Participants' ages were mainly between 10 and 18 years old, with only two studies focusing on university students (Ojala, 2012b; Sayal et al., 2016). The most frequent study design was a quantitative survey, but a multi-method approach was also frequently implemented. Nearly half of the studies concerned programs or interventions aimed at promoting climate change engagement, awareness, knowledge, beliefs, attitudes, communication, systems thinking, behaviors, or behavioral intentions (Flora et al., 2014; Hu and Chen, 2016; Sayal et al., 2016; Bissinger and Bogner, 2018; Bentz and O'Brien, 2019; Trott, 2019; Deisenrieder et al., 2020). This review also included two models (Busch et al., 2019; Ojala and Bengtsson, 2019) and five exploratory studies relating several variables with well-being and climate-friendly behavior (Ojala, 2012a,b, 2013; Stevenson and Peterson, 2015; Bentz and O'Brien, 2019).

### Findings Concerning the Research Questions

#### How Is Positive Youth Development Addressed in Empirical Studies Relating Adolescents and Young Adults With Climate Change?

None of the selected studies for the final review mentioned the concept of positive youth development. Among studies previously subjected to full-text analysis, only one included PYD as a keyword, but without further development (Kretser and Chandler, 2020). However, some similarities were found with PYD principles. The rationale behind some of the analyzed studies was the idea that youth are potential agents of social change, with an important role to play in climate change responses (Ojala, 2012b, 2013; Flora et al., 2014; Sayal et al., 2016; Bentz and O'Brien, 2019; Trott, 2019) but also profoundly affected by this global challenge (Ojala, 2012a; Stevenson and Peterson, 2015; Ojala and Bengtsson, 2019; Deisenrieder et al., 2020). Three of the selected papers simultaneously addressed individual and systemic dimensions. Ojala (2012a) and Ojala (2013) investigated how different coping strategies are associated with well-being, based on the premise that the way people cope with climate change threat could be important for environmental engagement and psychological well-being. Bentz and O'Brien (2019) explored how promoting reflection on relationships between individual change and systems change facilitates a better understanding of the social-ecological complexities of climate change and deeper awareness of human agency in this process. In addition, Trott (2019) stressed the importance of a positive approach focused on youth agentic capabilities, mentioning the importance of looking at young people as agents of change rather than focusing on their vulnerabilities.

#### Which PYD Constructs Are Included in These Empirical Studies?

Similar constructs were conceptualized differently by each author, and some were presented as components of broader concepts. All studies included at least two PYD constructs according to Catalano's (2019) categorization. PYD constructs in the assets domain included critical or systems thinking (Sayal et al., 2016; Bentz and O'Brien, 2019), coping strategies (Ojala, 2012a,b, 2013; Ojala and Bengtsson, 2019), and communication types with significant others (Ojala and Bengtsson, 2019). Systems thinking is conceptualized as a form of critical thinking (Sayal et al., 2016). Both types of thinking express an increased perception of how climate change is related to the interconnections of the social-ecological system. The reference to coping strategies is associated with acknowledging climate change as a stressor and the importance of cognitive and emotional dimensions of coping both for engagement and psychological well-being, including the well-being of others (Ojala, 2013). Eight articles included constructs related to agency, as self, collective or environmental efficacy (Ojala, 2012a, 2013; Flora et al., 2014; Busch et al., 2019; Deisenrieder et al., 2020), sense of agency (Trott, 2019), hope (Ojala, 2012b; Stevenson and Peterson, 2015), perceived behavioral control as a determinant of behavioral intentions (Hu and Chen, 2016), and locus of control as part of attitudes (Deisenrieder et al., 2020). Efficacy was commonly explored as a variable influencing environmental action and linked explicitly to locus of control (Deisenrieder et al., 2020) and implied in positive expectations about the future (Ojala, 2012b; Stevenson and Peterson, 2015). Constructs concerning contributions were mentioned in all but one study focused on how coping strategies regulate worry and promote hope (Ojala, 2012b). These mainly included (self-reported) pro-environmental, conservation, ecological, climate-friendly, or sustainable behavior (Stevenson and Peterson, 2015; Bissinger and Bogner, 2018; Bentz and O'Brien, 2019; Busch et al., 2019; Ojala and Bengtsson, 2019; Deisenrieder et al., 2020) or environmental engagement (Flora et al., 2014; Sayal et al., 2016; Trott, 2019). Specifically, two studies considered pro-environmental behavior (Ojala, 2012a, 2013) or behavioral intentions (Flora et al., 2014; Hu and Chen, 2016) as part of environmental engagement. Engagement in climate change is conceptualized as encompassing cognitive, affective, and behavioral aspects (Flora et al., 2014; Sayal et al., 2016; Trott, 2019). All of the mentioned types of behavior are related explicitly to mitigation of climate change and associated with specific actions as transportation choice (Stevenson and Peterson, 2015; Bentz and O'Brien, 2019), energy conservation, waste avoidance, and consumerism (Bissinger and Bogner, 2018; Busch et al., 2019), but also information-seeking behavior (Stevenson and Peterson, 2015). Finally, under the domain enabling environment, some constructs related to bonding were identified, namely inclusion of nature in one's self, representing connection and conservation tendencies toward nature (Bissinger and Bogner, 2018), and place attachment, particularly associated with cultivating emotional engagement (Hu and Chen, 2016). Social or subjective norms were addressed as exerting significant influence over youth decision-making (Busch et al., 2019).

#### How Do These Studies' Outcomes Inform Future Approaches to Promote Positive Youth Development in the Context of Climate Change?

Globally, the programs considered in this review were successful in their aims, whether promoting skills or climate engagement. Thus, attention was given to program features that may inform future approaches since some studies' rationale claimed the need for appropriate intervention beyond climate science knowledge (Sayal et al., 2016; Bissinger and Bogner, 2018; Busch et al., 2019; Deisenrieder et al., 2020). These comprised developing arts projects and adopting sustainable behaviors for some time (Bentz and O'Brien, 2019), simulating authentic environments in a botanical garden or an alpine setting (Bissinger and Bogner, 2018; Deisenrieder et al., 2020), developing an assembly combining educational and entertainment characteristics (Flora et al., 2014), promoting communication with seniors (Hu and Chen, 2016), involving international speakers on environmental justice and exchange between a developed and a developing country (Sayal et al., 2016), and engaging participants in youth-led programs (Trott, 2019). Deisenrieder et al. (2020) stressed that some environment-friendly actions are out of adolescents' scope. Thus, his measure of climate-friendly behavior comprised multiplicative action by influencing family and friends. In addition, these authors specifically explored differences between participants in the Fridays for Future movement and those who only took part in an intervention, finding that the first showed higher means in action-related components of climate change awareness. However, some authors recognize limitations while interpreting these results: no evaluation about whether changes are sustained over time (Sayal et al., 2016; Trott, 2019), no exploration of other potential explanatory variables (Deisenrieder et al., 2020), testing effects (Flora et al., 2014), lack of a control group (Flora et al., 2014; Deisenrieder et al., 2020) or interventions not aiming at promoting long-term changes in behaviors (Bentz and O'Brien, 2019). Moreover, unexpected results should be taken into consideration. One study mentioned some feelings of disempowerment after the intervention (Bentz and O'Brien, 2019). On a positive note, the same authors also reported influence on family and friends and continued behavioral changes. The role of family and friends was mentioned in two other studies. Flora et al. (2014) reported that the behaviors most influenced by the intervention are communication with family and friends, and Ojala and Bengtsson (2019) concluded that positive communication patterns with parents and friends were positively related to problem and meaning-focused coping strategies. In turn, models and exploratory studies analyzed within this review advanced empirical evidence concerning pro-environmental behavior or mitigation intentions predictors, such as social norms (Busch et al., 2019), perceived behavioral control (Hu and Chen, 2016), efficacy (Busch et al., 2019), norms and place attachment (Hu and Chen, 2016), problem and meaning-focused coping (Ojala, 2012a, 2013; Ojala and Bengtsson, 2019), and hope and higher socioeconomic status (Stevenson and Peterson, 2015). It was also found that efficacy mediated the effects of social norms on behavior (Busch et al., 2019) and was positively associated with problem and meaning-focused coping (Ojala, 2012a, 2013). However, only meaning-focused coping is associated with positive affect and some dimensions of well-being, life satisfaction, and positive affect (Ojala, 2012a), hope (Ojala, 2012b), and optimism (Ojala, 2012a, 2013).

## Discussion

### Positive Youth Development and Climate Change

This review aimed to explore how PYD theory is integrated within climate change literature and advance current knowledge. We may find justification for the scarce direct mentions of PYD in the fact that so far, studies concerning climate change have focused mainly on adults (Majeed and Lee, 2017; Burke et al., 2018; Sanson et al., 2019). Interest in this developmental period triggered by the youth climate movement is still novel. Also, as noticed in two recent systematic reviews (Monroe et al., 2019; Roussell and Cutter-Mackenzie-Knowles, 2020), research has placed a greater emphasis on climate education, mainly in top-down and science-based interventions. As Eichas et al. (2019) advanced, PYD is a demanding framework that requires significant methodological shifts if used as more than a guiding meta-theory.

However, this review advances similarities concerning PYD approaches and current climate change research. We have found four main ideas in common: (1) the focus on youth as agents of change, (2) the double target of promoting well-being and engagement, (3) the relevance of systemic thinking, and (4) program characteristics. Thus, some reflections can be made on how the PYD framework could enhance these features in the context of climate change. Firstly, agency is a core dimension of PYD (Lerner et al., 2002) and has been considered a central component of studies focused on promoting children and youth adaptation in the context of climate change (Sanson et al., 2019; Hickman et al., 2021). In this regard, it is important to mention that some authors (Walker, 2017; Börner et al., 2020; Trott, 2021) have recently introduced a more multifaceted understanding of agency in the context of climate change action, which comes across with the ecological features of PYD approaches. This notion is based on everyday interaction with the environment, focusing research on the capacity and potential of youth as everyday agents and young citizens and not necessarily engaged in more visible forms of agency. Secondly, the holistic and integrative approach provided by the PYD framework can tackle both well-being and engagement. Within this review, well-being dimensions have been mostly associated with meaning-focused coping strategies that imply activating positive feelings and values to buffer negative feelings and sustain well-being and positive action (Ojala, 2012a,b, 2013). This type of strategy has been recently discussed under different conceptualizations in climate change literature concerning eco-anxiety (Clayton and Karazsia, 2020; Hickman, 2020; Hickman, 2021) and eco-anger (Stanley et al., 2021). We believe that the positive focus brought by positive development constructs (Tolan, 2014) could help support the integration of these feelings. Engagement with climate issues, in turn, is mainly associated with climate-friendly behaviors in the studies included in this review. Behaviors are considered a priority to tackle by Psychology and the social sciences (Nielsen et al., 2021). These authors suggest that it is not sufficient to consider behavior plasticity but also behaviors impact and feasibility considering surrounding contexts and the multiplicity of roles played by the individuals. As discussed in previous sections, we argue that PYD ecological foundations could bring new input to these desirable pathways on climate-related behaviors research. A relevant idea highlighted within this review is that some environment-friendly actions are out of adolescents' scope, who can anyway have a multiplicative action by influencing family and friends (Trott, 2019). We remind that PYD adds the possibility of cascade effects since additional outcomes may be expected from PYD interventions than the targeted ones (Eichas et al., 2019). This means that PYD interventions may result in many positive outcomes for participants with different characteristics. Thirdly, in this review and the broader context of climate change research, a systemic approach has been recommended, acknowledging the diverse contexts and their reciprocities when considering climate change issues (Berry et al., 2018). Including multiple contexts to inform developmental trajectories is precisely one of PYD's major strengths (Benson et al., 2007; Sherrod, 2007). Finally, we have noticed that some PYD programs characteristics, as posited by Roth and Brooks-Gunn (2003) and Lerner (2004), namely real-life experiences, authentic environments, opportunities for a proactive role, and significant interactions with meaningful adults, were included in some studies as an attempt to go further than traditional science-based interventions.

### Strengths and Limitations

As the main strength of this review, we highlight the fact of opening space to a different approach concerning current research about adolescents and young adults in the context of climate change. Developmental science is positioned as a relevant and transdisciplinary contributor to climate change studies. In addition, we add evidence to the potential that has been acknowledged to Positive Youth Development models in the context of global crises. Nevertheless, some limitations must be considered. We note that relevant studies may not have been included in this review due to strict inclusion criteria: the exclusive focus in English, in peer-reviewed articles, and on studies that exclusively concern the broad concept of climate change. Additionally, constraints inherent to selecting specific databases and the search terms may have led to missing some studies. Finally, even though it might have resulted in additional relevant information, this review did not intend to provide a thorough analysis of program efficacy or comparison between studies. It would be anyhow a challenging task, considering both PYD (Tolan, 2014; Ciocanel et al., 2017; Leman et al., 2017; Lerner et al., 2018; Shek et al., 2019) and climate change studies (Sanson et al., 2018) lack a broadly accepted standard structure to allow comparisons.

### Implications for Policy, Practice, and Future Research

Within this review, we have gathered some evidence that PYD can be an adequate approach to be considered by policymakers, researchers, and practitioners. A PYD perspective may open the way to a new age of more developmental and bioecologically sensitive approaches, tackling at the same time two purposes. On the one hand, this framework promotes specific skills that are useful to climate change engagement. Furthermore, it facilitates the development of more globally competent and adjusted individuals who contribute significantly to their own lives and society. We find this argument particularly relevant for investors and policymakers. Furthermore, even though we acknowledge a psychological perspective, we are aware of the interdisciplinarity required concerning climate change research (Nielsen et al., 2021). This review has shown that social sciences and life sciences researchers co-authored a significant part of the included papers. PYD theory already surpasses this demand, as it is known by its bridging character, which crosses diverse academic domains and multiple spheres of practice (Benson et al., 2007).

Additionally, as described in the previous sections, we have provided some clues about the most relevant constructs and features to consider in future interventions and several variables found to be positively related to pro-environmental behaviors. Considering the systemic and ecological features of the climate change phenomenon, we suggest that some of the variables found in this review reflecting bonding with place and nature should also be considered in future studies. Literature refers that becoming bonded to a place has psychological benefits and implications for climate. If someone has a strong attachment to a place, they probably want to protect it (Scannell and Gifford, 2013; Gifford, 2014). Regarding program evaluation, we suggest that search for evidence of alignment concerning individual-context relations should be added to group differences analysis as an indicator of intervention success in PYD programs, as posited by Tolan (2016).

Concerning future research, we find relevant a deeper understanding of the individual-systems interrelations in the context of climate change. Developmental researchers have long advocated for more research on everyday contexts (Dahl, 2017). This could be achieved by exploring how adolescents and young adults think, feel, and act facing the climate threat and how this may affect well-being and influence behaviors and its impact and feasibility. Daily life studies may be informative of these interconnections and add data to a more multifaceted understanding of agency, studying youth potentialities as everyday agents and not exclusively on acknowledged forms of activism. This type of study would also allow both an interindividual and intraindividual analysis. Feelings of disempowerment should be carefully analyzed as a possible result of interventions concerning climate change. In addition, a significant gap was detected concerning research among young adults. Considering the broad scope of adolescence's current conceptualization (Sawyer et al., 2018) and PYD specificities across developmental periods, studying differences according to each age group would also be relevant. Finally, given that PYD constructs may be manifested differently in diverse cultural contexts (Lerner et al., 2018), we acknowledge the need for further studies concerning countries out of American and European countries.

## Conclusions

We conclude that PYD theory is not yet deliberately integrated into studies concerning adolescents and young adults in the context of climate change. However, this review's search for common denominators demonstrates that several of its constructs and principles are acknowledged within current research. A strong intersection has been identified, and this provides innovating clues and a pathway for future research. In a moment in which the international scientific community requires insights from the social sciences, particularly psychology, to contribute to achieving climate change targets (cf. Nielsen et al., 2021), we advocate for PYD as an innovative and promising approach. PYD offers a multidisciplinary, comprehensive, and holistic perspective aligned with climate change research requirements. We also notice that an enquiring word parallel may be found with Nationally Determined Contributions (NDC, Falkner, 2016). These national plans regarding climate actions are currently considered the crucial means to strengthen the global response to the threat of climate change. This review highlights the perspective that youth contribution has been essential to mobilize international action and will be fundamental to sustain climate change targets in the future. The investment in youth development is, thus, a priority. It is essential to promote adaptability, attenuate expected or already vivid impacts from climate change, and support adolescents and young adults' active engagement. Finally, we believe in having encouraged a step forward regarding developmental psychology acknowledgment in the context of climate change research. As previously asserted (Gauvain, 2018), developmental scientists' involvement may contribute to the effectiveness of many projects and new reflections regarding youth policies. In addition, by refocusing research on current global changes and significant consequential stresses in youth lives, development theory may be enriched with new insights regarding adolescents' and young adults' current experiences, challenges, and resilience.

## Data Availability Statement

The original contributions presented in the study are included in the article/supplementary material, further inquiries can be directed to the corresponding author/s.

## Author Contributions

TP wrote the first draft of the manuscript. TF critically revised it. All authors contributed to the study's conception and design, performed the literature search and data analysis, and read and approved the final manuscript.

## Funding

This study was conducted at the Psychology Research Centre (PSI/01662), School of Psychology, University of Minho, supported by the Foundation for Science and Technology (FCT) through the Portuguese State Budget (UIDP/PSI/01662/2020) and under a Ph.D. fellowship also supported by the FCT (SFRH/BD/143814/2019).

## Conflict of Interest

The authors declare that the research was conducted in the absence of any commercial or financial relationships that could be construed as a potential conflict of interest.

## Publisher's Note

All claims expressed in this article are solely those of the authors and do not necessarily represent those of their affiliated organizations, or those of the publisher, the editors and the reviewers. Any product that may be evaluated in this article, or claim that may be made by its manufacturer, is not guaranteed or endorsed by the publisher.
